# Diagnostic value of artificial intelligence-assisted CTA for the assessment of atherosclerosis plaque: a systematic review and meta-analysis

**DOI:** 10.3389/fcvm.2024.1398963

**Published:** 2024-09-03

**Authors:** Pingping Jie, Min Fan, Haiyi Zhang, Oucheng Wang, Jun Lv, Yingchun Liu, Chunyin Zhang, Yong Liu, Jie Zhao

**Affiliations:** ^1^ Department of Magnetic Resonance Imaging, The Affiliated Traditional Chinese Medicine Hospital, Southwest Medical University, Luzhou, China; ^2^ Department of Radiology, The Affiliated Traditional Chinese Medicine Hospital, Southwest Medical University, Luzhou, China; ^3^ Department of Comprehensive Internal Medicine, The Affiliated Chinese Traditional Medicine Hospital, Southwest Medical University, Luzhou, China; ^4^ Department of Nuclear Medicine, The Affiliated Hospital of Southwest Medical University, Luzhou, China; ^5^ Department of Nuclear Medicine and Molecular Imaging Key Laboratory of Sichuan Province, The Affiliated Hospital, Southwest Medical University, Luzhou, China

**Keywords:** AI, CTA, plaque, assessment, meta-analysis, systematic review

## Abstract

**Background:**

Artificial intelligence (AI) has increasingly been applied to computed tomography angiography (CTA) images to aid in the assessment of atherosclerotic plaque. Our aim was to explore the diagnostic accuracy of AI-assisted CTA for plaque diagnosis and classification through a systematic review and meta-analysis.

**Methods:**

A systematic literature review was performed by searching PubMed, EMBASE, and the Cochrane Library according to PRISMA guidelines. Original studies evaluating the diagnostic accuracy of radiomics, machine-learning, or deep-learning techniques applied to CTA images for detecting stenosis, calcification, or plaque vulnerability were included. The quality and risk of bias of the included studies were evaluated using the QUADAS-2 tool. The meta-analysis was conducted using STATA software (version 17.0) to pool sensitivity, specificity, and area under the receiver operating characteristic curve (AUROC) to determine the overall diagnostic performance.

**Results:**

A total of 11 studies comprising 1,484 patients were included. There was low risk of bias and substantial heterogeneity. The overall pooled AUROC for atherosclerotic plaque assessment was 0.96 [95% confidence interval (CI) 0.94–0.97] across 21 trials. Of these, for ≥50% stenosis detection, the AUROC was 0.95 (95% CI 0.93–0.96) in five studies. For identifying ≥70% stenosis, the AUROC was 0.96 (95% CI 0.94–0.97) in six studies. For calcium detection, the AUROC was 0.92 (95% CI 0.90–0.94) in six studies.

**Conclusion:**

Our meta-analysis demonstrates that AI-assisted CTA has high diagnostic accuracy for detecting stenosis and characterizing plaque composition, with optimal performance in detecting ≥70% stenosis.

**Systematic Review Registration:**

https://www.crd.york.ac.uk/, PROSPERO, identifier (CRD42023431410).

## 1 Introduction

Cardiovascular disease (CVD) and acute cerebrovascular disease (ACD) have become leading causes of mortality and morbidity globally (1–3), with most CVD- and ACD-related deaths attributable to acute myocardial infarction and ischemic stroke (4, 5), which was related to atherosclerotic plaque rupture or erosion (6, 7). Accurate plaque characterization is essential for clinical decision-making in patients with atherosclerosis, such as degree of stenosis (8), composition (9), vulnerability (10), and other characteristics (11), which are critical for the treatment of patients with atherosclerosis (9, 12) and will facilitate developing appropriate treatment regimens. Computed tomography angiography (CTA) has become a vital non-invasive imaging modality for comprehensive plaque evaluation (13), enabling quantification of stenosis, delineation of morphology, and characterization of high-risk features associated with increased plaque rupture risk (9, 14). However, this complex postprocessing and measurement relies on operator experience, which has plagued radiologists (14).

Recently, artificial intelligence (AI) has exhibited remarkable progress attributable to advances in computing power and deep-learning algorithms (15). One of the most exciting fields in medicine for AI applications is radiology medicine. There are numerous AI applications in cerebral and cardiovascular imaging with the potential to automate laborious image analysis tasks, extract additional diagnostic and prognostic insights beyond human interpretation, and optimize workflow efficiency (16). Moreover, AI has the ability to automate image processing, draw out more therapeutically useful insights from images, and forecast the likelihood of prognostic outcomes. Numerous studies have demonstrated that AI-based systems are effective in diagnosing a wide range of illnesses (17–19). Machine learning has shown promising results in automatically identifying and excluding coronary stenoses on coronary CTA, and AI-based assessments were highly accurate for severe stenoses at the ≥50% and ≥70% levels. Therefore, AI-assisted CTA image analysis may serve as a powerful tool to augment plaque quantification and characterization (15).

Over the last few years, AI-based algorithms and automated software platforms have been developed for plaque evaluation (20–24). However, there are discrepancies across different studies. Therefore, we conducted a systematic review and meta-analysis of published studies to critically evaluate the diagnostic accuracy of AI-assisted CTA in detecting stenosis and characterizing plaque composition and vulnerability features compared to reference standards.

## 2 Methods

The protocol for this systematic review and meta-analysis was prospectively registered on PROSPERO (registration number CRD42023431410). The systematic review and meta-analysis was conducted in accordance with the Preferred Reporting Items for Systematic Reviews and Meta-Analyses (PRISMA) guidelines (25) to ensure robust methodology and comprehensive reporting. Data analysis adhered to recommendations from the Cochrane Handbook for Diagnostic Test Accuracy Reviews (26).

### 2.1 Search strategy

A comprehensive literature search was conducted in the PubMed, Embase, and Cochrane Library databases up to 8 July 2023, incorporating MeSH terms and free-text keywords related to “Artificial Intelligence,” “Machine Learning,” “Deep Learning,” “Radiomics,” “Computed Tomography Angiography,” and “Plaque.” The purpose of this search strategy was developed to identify all studies assessing the diagnostic performance of AI-assisted CTA for plaque analysis. The strategy included a wide range of relevant literature without restrictions based on publication date or language to avoid bias. The process was refined iteratively to ensure a comprehensive overview of the evidence base on AI-assisted CTA for plaque analysis.

### 2.2 Inclusion and exclusion criteria

Studies were selected for inclusion after removing duplicates if they met the following criteria: (1) patients with coronary or carotid plaque were included in study; (2) artificial intelligence algorithms based on CT images was applied to evaluate the diagnostic accuracy; (3) at least 10 patients were included; and (4) the true-positive (TP), false-positive (FP), false-negative (FN), and true-negative (TN) rates could be calculated from the data.

Studies were excluded if they were: (1) personal communications, editorials, letters, abstracts, conferences, or case reports; (2) not using AI-assisted CTA images; (3) not investigating humans, but for experimental animals; or (4) non-English publications. Two reviewers (PJ and MF) independently screened all identified studies against the eligibility criteria, extracted the relevant data, and assessed the study quality. Any disagreements were resolved by discussion with a third reviewer (JZ).

### 2.3 Data extraction

Two reviewers independently extracted the following key data from eligible studies using standardized forms: (1) study details: publication year, country, design, sample size, title, population demographics, imaging used; (2) AI algorithm information: imaging modality, feature extraction techniques, algorithm type and name; (3) diagnostic performance metrics: area under the receiver operating characteristic curve (AUROC), sensitivity (SEN), specificity (SPE), TP, FP, FN, and TN.

### 2.4 Assessment of study quality

The quality assessment of all eligible studies was performed independently using the revised Quality Assessment Tool for Diagnostic Accuracy Studies (QUADAS)-2 (27). This comprehensive assessment encompassed four key domains, namely “Patient Selection,” “Index Test,” “Reference Standard,” and “Flow and Timing” to ascertain potential biases, with the first three domains also undergoing scrutiny for concerns relating to their applicability. Quality ratings were assigned as “high,” “low,” or “unclear.” Discrepancies in the assessment process were resolved through consensus between two reviewers (PJ and MF). Furthermore, the bias risk for each included study was evaluated using the Review Manager 5.3 software to facilitate an in-depth analysis of the quality of diagnostic articles.

### 2.5 Data and statistical analysis

The meta-analysis and statistical analysis were conducted following rigorous adherence to the established Cochrane guidelines. The presence of the threshold effect was assessed using the spearman correlation coefficient, with a *p*-value <0.05 serving as an indicator of its presence (28). Moreover, the evaluation of statistical heterogeneity was carried out using the *I*^2^ statistics (26), The value of *I*^2^ exceeding 50% was conventionally considered to indicate substantial heterogeneity, while a value of *I*^2^ below 50% suggested a lower degree of heterogeneity (29). Pooled estimates of SEN, SPE, positive predictive value (PPV), negative predictive value (NPV), and diagnostic odds ratio (DOR) were computed using a random effects model. The derivation of these estimates involved the integration of the raw data on TP, FP, TN, and FN values reported in each study. The certainty of these pooled estimates was further substantiated through the calculation of 95% confidence intervals (95% CIs) (30). In addition, the AUROC was constructed, a graphical representation that encapsulates the diagnostic performance of the AI-assisted system across various threshold settings. The curve allowed for the calculation of the area under the curve (AUC), a pivotal metric was used to quantify the overall diagnostic accuracy. Notably, different ranges of AUROC values (0.5–0.7, 0.7–0.9, and 0.9–1) were utilized to categorize the diagnostic accuracy as low, moderate, and high, respectively (31). Funnel plots were used to assess articles for publication bias. The entire meta-analysis procedure was carried out utilizing the Meta-DiSc 1.4 and “MIDAS” modules within the STATA 17 software (version 17.0 IC; Stata Corporation, College Station, TX, USA). Furthermore, a comprehensive subgroup analysis was implemented to elucidate potential sources of heterogeneity, encompassing various factors, such as arterial region (carotid or coronary artery), study design (prospective or retrospective), the geographic origin of the literature (Asia, Europe, or America), AI methodology (deep learning or machine learning), and study center type (multicenter or single-center). This approach allowed for a thorough exploration of potential variations in the diagnostic accuracy of AI-assisted systems, providing valuable insights into the underlying factors influencing the observed heterogeneity.

## 3 Results

### 3.1 Selection of studies

**Figure 1:**
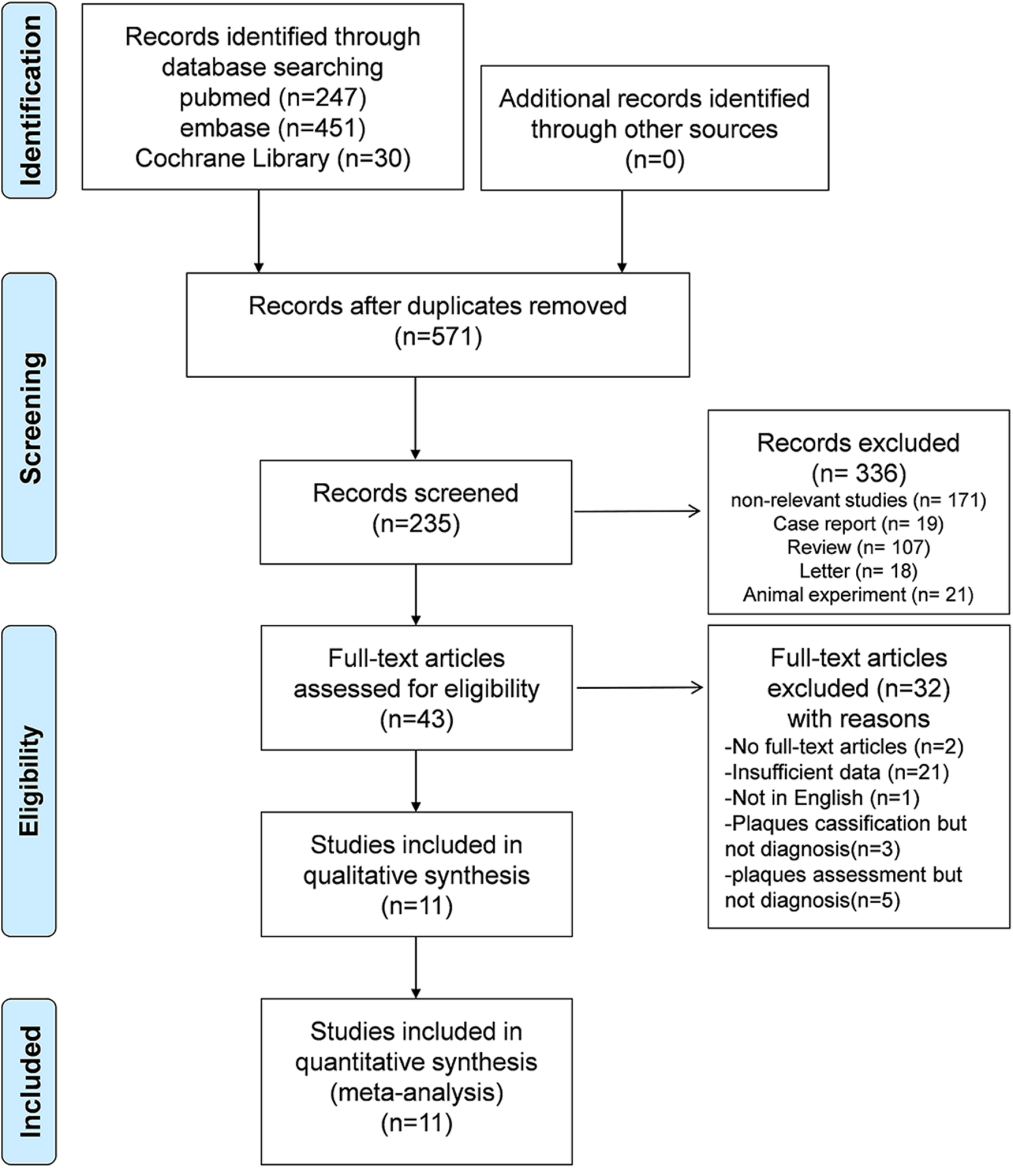
Flow diagram of the study selection process for this review.

The literature search and screening process are presented in Figure 1. A total of 728 potentially eligible articles were initially identified through the database search. After the exclusion of 157 duplicates, 571 articles were subjected to screening based on titles and abstracts, resulting in the selection of 43 studies for full-text review. Ultimately, 11 articles pertaining to AI-assisted CTA diagnostic technology for plaque assessment were incorporated into this meta-analysis (20–24, 32–37), and the comprehensive details of the literature search and screening process are depicted in Figure 1.

### 3.2 Study characteristics

Table 1 and Supplementary Table S2 provide a detailed overview of the characteristics of the 11 identified studies comprising 21 trials, with publication dates between 2019 and 2023. The cumulative number of participants across all studies was 1,484, with a wide age range of 33–88 years. Five studies containing 15 trials investigated vessel stenosis caused by plaque, of which 8 trials focused on 50% stenosis and 7 trials on 70% stenosis. Five studies containing five trials investigated calcified plaque and one remaining study investigated plaque vulnerability. Nine studies containing 16 trials investigated coronary plaque and 2 remaining studies containing 5 trials examined carotid plaque. Four studies containing 12 trials were multicenter studies while the remaining 7 studies containing 9 trials were single-center studies. In terms of the applied algorithm, four studies focused on deep learning, five studies used machine learning, and artificial intelligence was used in two studies. Semi and automatic segmentation were applied in nine studies, whereas manual segmentation of images was reported in two studies. The systematic review showed that AI-assisted CTA diagnostic technology for the assessment of plaque had SEN rates in the range of 64.00%–100.00%, SPE rates in the range of 43.90%–99.80%, and AUC in the range of 69.00%–96.00%.

**Table 1:** Main characteristics of the included studies.

Author	Year	Country	Mean or median age	Patients	AI model	Centers^a^	Plaque classification
Acharya	2019	Singapore	60.7 ± 10.4	73	Machine learning	S	Calcified plaque
Han	2020	China	64.0	50	Deep learning	S	Calcified plaque
Li	2021	China	53.0 ± 9.0	36	Machine learning	S	Vulnerable atherosclerotic
Choi	2021	USA	60.0 ± 12.0	232	Artificial intelligence	M	>70% stenosis
Choi	2021	USA	60.0 ± 12.0	232	Artificial intelligence	M	>50% stenosis
Choi	2021	USA	60.0 ± 12.0	232	Artificial intelligence	M	>70% stenosis
Choi	2021	USA	60.0 ± 12.0	232	Artificial intelligence	M	>50% stenosis
Xu	2021	China	65.7 ± 10.1	306	Artificial intelligence	S	>50% stenosis
Xu	2021	China	65.7 ± 10.1	306	Artificial intelligence	S	>70% stenosis
Xu	2021	China	65.7 ± 10.1	306	Artificial intelligence	S	>50% stenosis
Yi	2021	China	63.3 ± 10.7	71	Deep learning	S	Calcified plaque
Lin	2022	USA	—	100	Deep learning	M	>70% stenosis
Lin	2022	USA	—	100	Deep learning	M	>50% stenosis
Griffin	2022	USA	64.0 ± 10.0	303	Machine learning	M	>50% stenosis
Griffin	2022	USA	64.0 ± 10.0	303	Machine learning	M	>70% stenosis
Cilla	2022	Italy	73.0	30	Machine learning	S	Hard plaque
Hu	2022	China	—	141	Machine learning	S	Calcified plaque
Fu	2023	China	61.0 ± 11.0	142	Deep learning	M	>70% stenosis
Fu	2023	China	61.0 ± 11.0	142	Deep learning	M	>50% stenosis
Fu	2023	China	61.0 ± 11.0	142	Deep learning	M	>70% stenosis
Fu	2023	China	61.0 ± 11.0	142	Deep learning	M	>50% stenosis

^a^ S, single-center; M, multicenter.

### 3.3 Quality assessment of the studies

We employed the radiomics quality score (RQS) (38) to assess the quality of the eligible studies. Supplementary Table S3 displays the mean score for each criterion of the RQS across all included studies. The mean RQS score of the studies was determined to be 24.7% [8.9 points, standard deviation (SD) ± 7.9 points], with a median score of 22.2% (8.0 points) and a range of 2.8%–72.2% (1–26 points). Notably, an upward trend in the RQSs of the included studies over time was observed, as depicted in Supplementary Figure S3.

**Figure 2:**
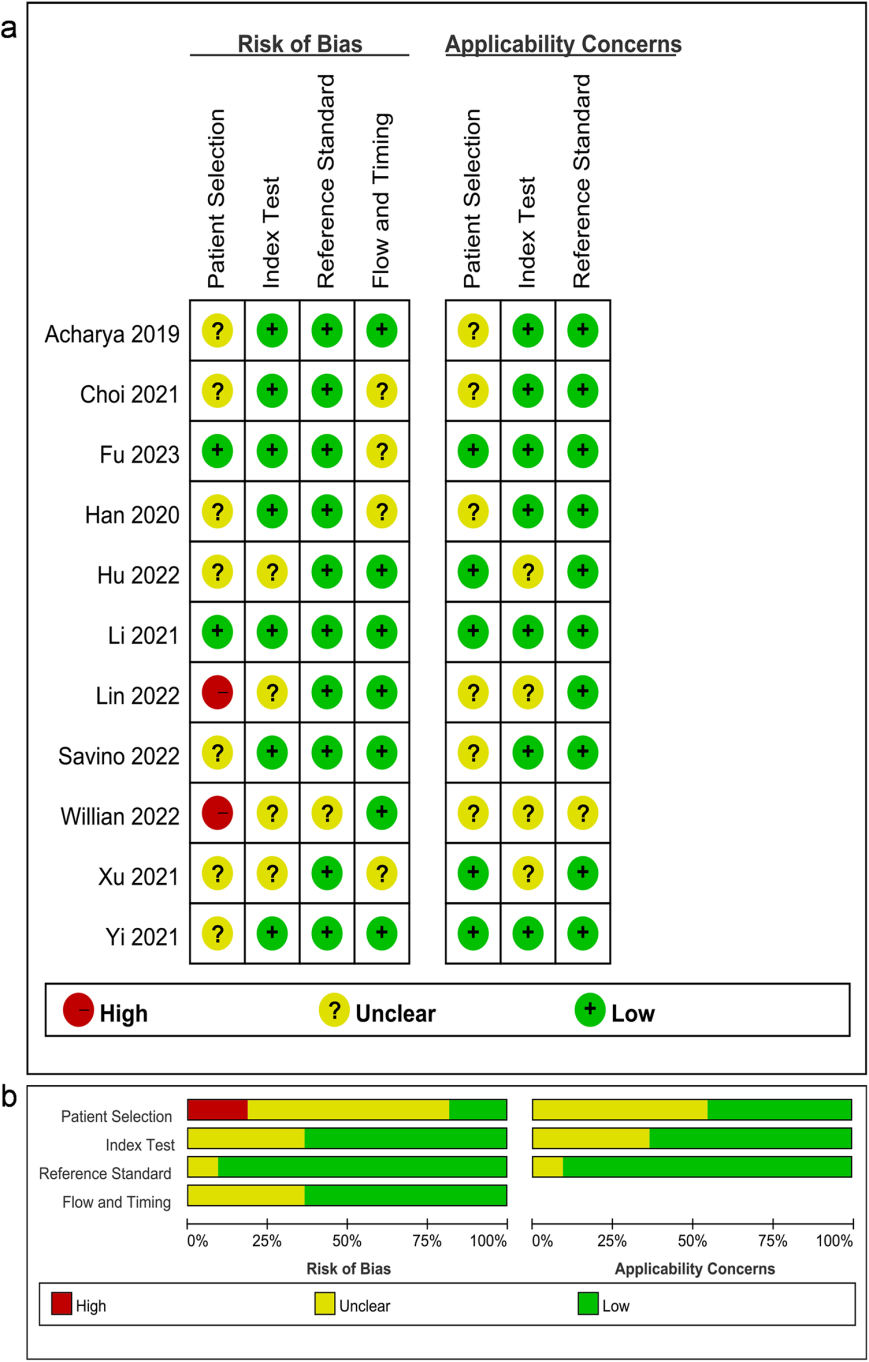
**(A)** Summary of the risk of bias and applicability concerns and **(B)** graph showing the quality of included studies according to QUADAS-2. Green represents low risk, yellow circle represents unclear risk, and red represents high risk of bias.

To evaluate the risk of bias and applicability concerns, we applied the QUADAS-2 tool (Figure 2). Concerning patient selection, two (18.18%) studies were deemed to possess a high risk of bias, whereas two (18.18%) studies were classified as low risk and seven (63.64%) studies were characterized by an unclear risk of bias. In the index test domain, seven (63.67%) studies were rated as low risk, while four (36.36%) studies were designated as having an unclear risk of bias. Regarding the reference standard domain, 10 (90.91%) studies were identified as low risk, with 1 (9.09%) study demonstrating an unclear risk of bias. As for flow and timing, four (36.36%) studies were classified as low risk and seven (63.67%) studies were regarded as having an unclear risk of bias.

The analysis revealed a notable risk of bias in the domain of patient selection, with the absence of randomization serving as a primary contributing factor. Overall, the included studies were deemed suitable for subsequent analyses in light of its quality.

### 3.4 Meta-analysis

**Figure 3:**
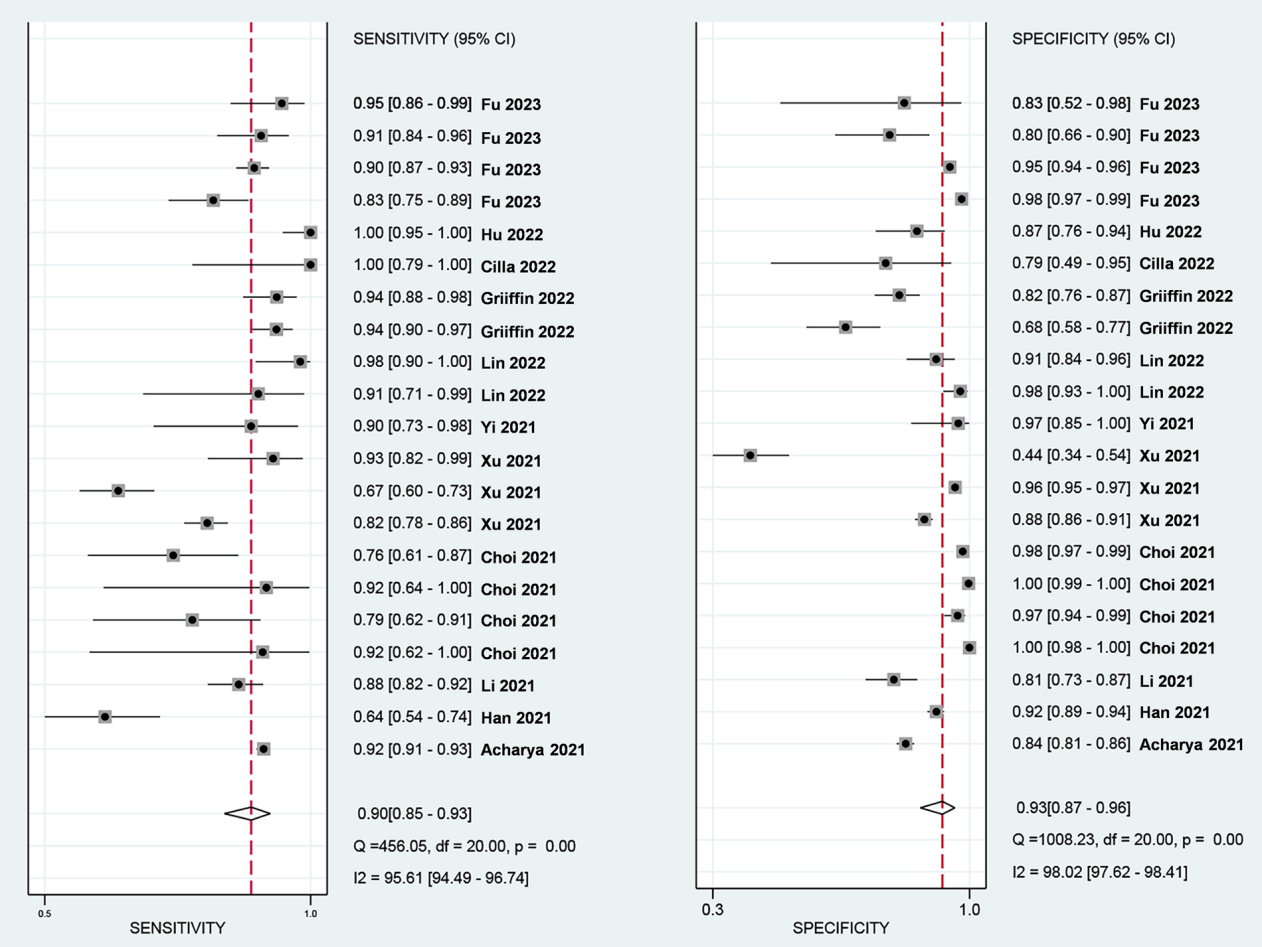
Forest plots of the pooled SEN (left) and SPE (right) for the diagnostic performance of AI-assist CTA for the assessment of atherosclerosis plaque.

**Figure 4:**
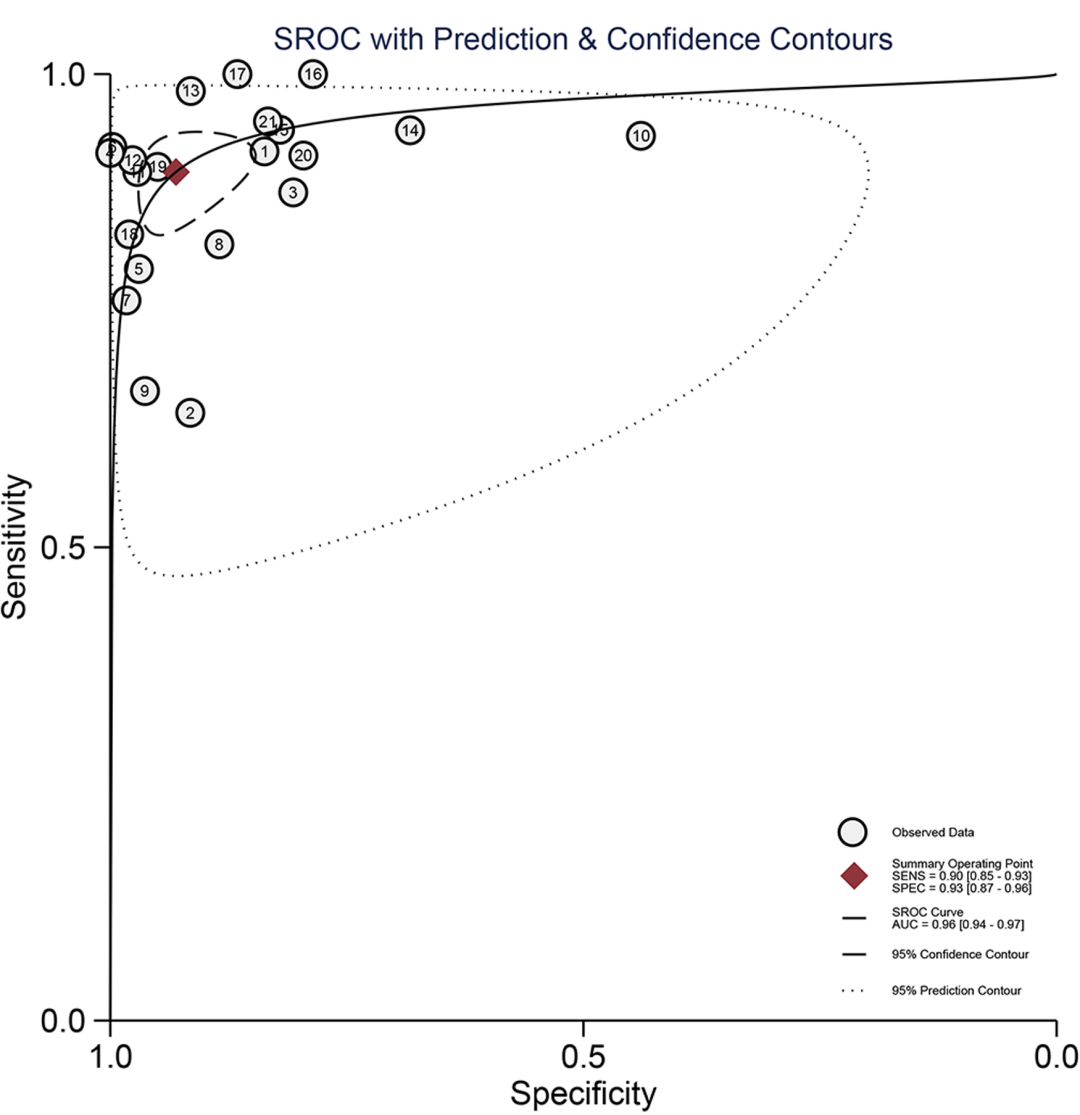
The pooled hierarchical summary receiver operating characteristic curve (AUROC) plot for the diagnostic performance of AI-based CTA for the assessment of atherosclerosis plaque.

The Spearman correlation coefficient was 0.418 (*p* = 0.201 > 0.05), suggesting that there was no discernible threshold effect for using AI-based CTA to evaluate atherosclerotic plaque. High heterogeneity was shown by the *I*^2^ values, which were 95.61% and 98.02% for pooled SEN and SPE, respectively (Figure 3). For evaluating atherosclerotic plaque using AI-based CTA, the pooled SEN and SPE were 0.90 (95% CI 0.85–0.93) and 0.93 (95% CI 0.87–0.96), respectively (Figure 3). The results showed that the AUROC was 0.96 (95% CI 0.94–0.97) (Figure 4 and Supplementary Table S4), which indicated a high diagnostic performance. In identifying ≥50% stenotic vessels, the SEN, SPE, and AUROC were 0.90 (95% CI 0.84–0.94), 0.89 (95% CI 0.76–0.96), and 0.95 (95% CI 0.93–0.96). In identifying ≥70% stenotic vessels, the SEN, SPE, and AUROC were 0.87 (95% CI 0.78–0.93), 0.98 (95% CI 0.91–0.99), and 0.96 (95% CI 0.94–0.97), which indicated high diagnostic accuracy. In identifying calcified plaque, the SEN, SPE, and AUROC were 0.94 (95% CI 0.73–0.99), 0.87 (95% CI 0.81–0.92), and 0.92 (95% CI 0.90–0.94), respectively (Supplementary Figure S2).

### 3.5 Publication bias

**Figure 5:**
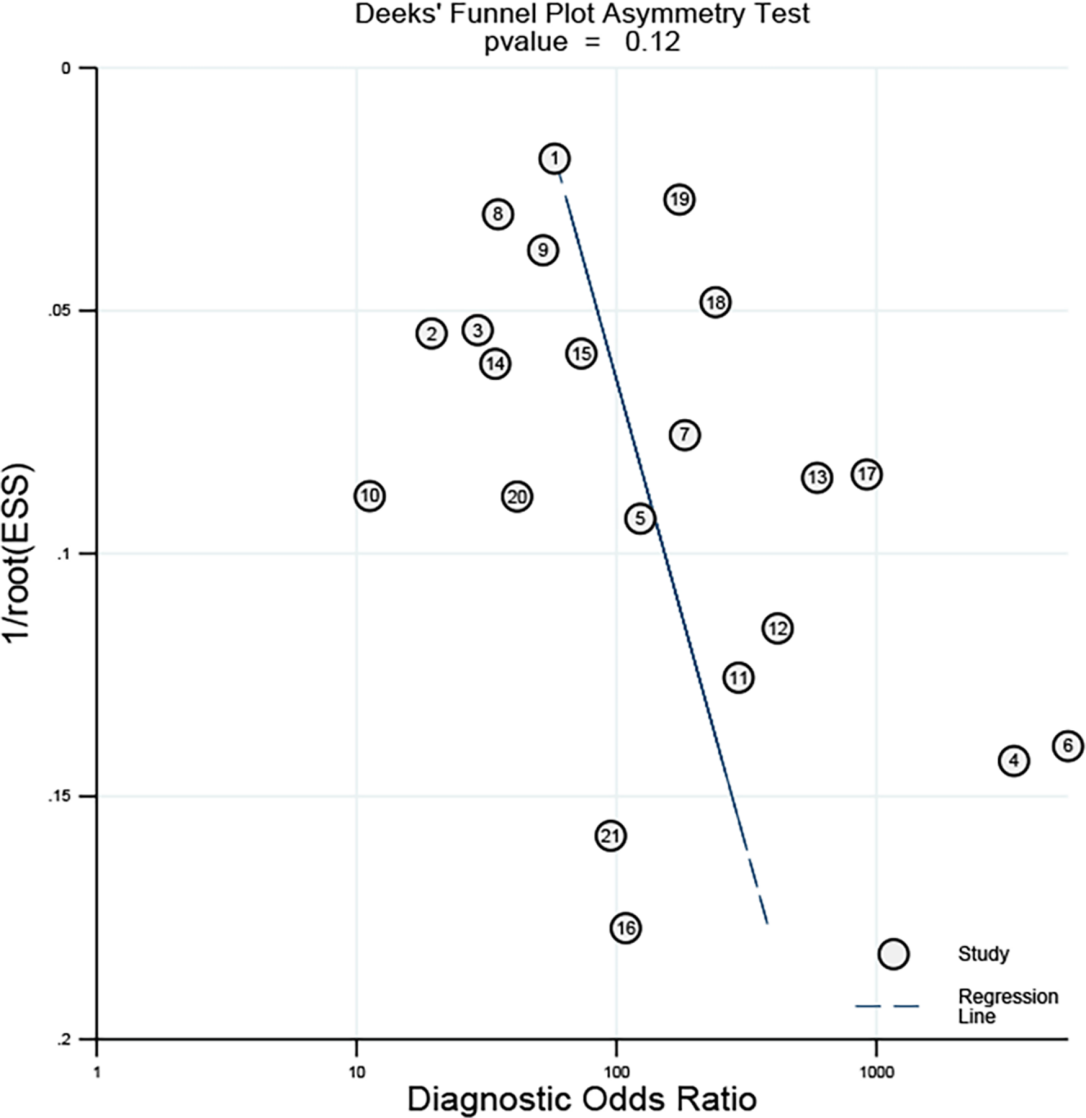
Deek’s funnel plot asymmetry test for assessment of publication bias.

As shown in Figure 5, Deek's funnel plot showed a *p*-value of 0.12, suggesting no obvious publication bias was found in all eligible studies.

### 3.6 Subgroup analysis

Table 2 presents the detailed outcomes of the subgroup analyses conducted to investigate the potential sources of heterogeneity. A slight drop in *I*^2^ was observed in SEN (from 95.61% to 76.57%) and SPE (from 98.02% to 94.80%) in the carotid plaque group after grouping according to whether carotid or coronary plaque was detected. After grouping according to the study design, a drop in *I*^2^ was observed in SEN (from 95.61% to 84.61%), suggesting the prospective study may reduce the probability of heterogeneity. Studies with a multicenter study design yielded a higher AUROC (0.96 vs. 0.86) and DOR (175 vs. 50) than those with a single-center study design. Further, utilizing machine- and deep-learning models to assist the diagnosis of atherosclerotic plaque has a higher SPE (0.92 vs. 0.81) and AUROC (0.96 vs. 0.90).

**Table 2:** Results of the subgroup analysis.

Analysis	No. of trials	No. of patients	SEN	*I*^2^ (%)	SPE	*I*^2^ (%)	DOR	AUROC
Overall group	21	1,484	0.90 (0.85–0.93)	95.61	0.93 (0.87–0.96)	98.02	116 (61–222)	0.96 (0.94–0.97)
Imaging artery								
Carotid	5	172	0.92 (0.87–0.95)	76.57	0.91 (0.80–0.96)	94.80	120 (66–220)	0.96 (0.94–0.98)
Coronary	16	1,312	0.89 (0.83–0.93)	97.37	0.94 (0.87–0.97)	98.86	124 (53–292)	0.96 (0.94–0.97)
Study design								
Prospective + retrospective	10	474	0.87 (0.82–0.91)	84.61	0.97 (0.93–0.99)	94.97	224 (129–390)	0.96 (0.94–0.97)
Retrospective	11	1,010	0.90 (0.83–0.94)	95.47	0.84 (0.75–0.90)	97.90	48 (28–82)	0.94 (0.91–0.95)
Region								
Asia	12	819	0.89 (0.82–0.93)	94.83	0.89 (0.82–0.94)	98.00	65 (35–118)	0.95 (0.92–0.96)
USA and Europe	9	665	0.91 (0.84–0.95)	94.73	0.96 (0.88–0.99)	97.76	264 (86–814)	0.96 (0.94–0.98)
Modeling methods								
Artificial intelligence	7	538	0.81 (0.71–0.88)	96.71	0.97 (0.87–0.99)	99.45	158 (29–868)	0.90 (0.87–0.92)
Machine/deep learning	14	946	0.92 (0.88–0.95)	91.89	0.90 (0.84–0.93)	95.85	100 (55–179)	0.96 (0.94–0.98)
Center								
Single	9	707	0.89 (0.79–0.95)	96.04	0.86 (0.76–0.92)	98.31	50 (24–105)	0.94 (0.91–0.96)
Multiple	12	777	0.89 (0.85–0.92)	88.29	0.96 (0.90–0.98)	97.59	175 (96–318)	0.95 (0.93–0.97)

## 4 Discussion

In this study, a meta-analysis of the diagnostic accuracy of AI-assisted CTA for plaque diagnosis was performed. A total of 11 studies comprising 21 trials including 1,484 patients were included in this study. To assess risk of bias and applicability issues, we used the QUADAS-2 tool. Only two studies were considered to be at high risk of bias in terms of patient selection, and we therefore considered the quality of the study included in this study to be credible for use in a subsequent meta-analysis. RQSs showed that the median score of eligible studies was 22.2%. Interestingly, we found that RQSs got higher over time, suggesting that the quality of the literature may have been improved due to the development of AI technology. The results showed that the pooled SEN of this study was 0.90 (95% CI 0.85–0.93) and SPE was 0.93 (95% CI 0.87–0.96). The pooled AUROC of this study was 0.96 (95% CI 0.94–0.97), which indicated that AI-assisted CTA has a high diagnostic accuracy for assessing atherosclerotic plaque. A previous systematic review compared different imaging modalities using a radiomics feature for carotid plaque assessment. However, they did not perform a meta-analysis to show the pooled results because of the relatively small number and variations in the quality of the included articles (39). To the best of our knowledge, this is the first systematic review and meta-analysis of AI methods based on CTA images for plaque assessment, which is important for the identification and classification of vascular plaque. This meta-analysis showed high pooled SEN and SPE values of 0.90 (95% CI 0.85–0.93) and 0.93 (95% CI 0.87–0.96), respectively, which demonstrated that AI methods have the potential ability to accurately evaluate the plaques. Through our meta-analysis, the results showed that AI-assisted CTA had a relatively high AUROC of 0.96 (95% CI 0.94–0.97) for assessing plaque, which was higher than that in most similar articles.

CTA has the capacity to detect not only atherosclerotic plaque with its luminal narrowing but also its composition and morphology (40). The adoption of AI in CTA may greatly achieve a better diagnostic performance (40–42). In this article, we further evaluated the ability of AI in the identification and classification of vascular plaque. For the identification of stenosis, our results showed that the sensitivity of AI-assisted CTA was higher than the sensitivity in the non-radiomics method in diagnosing calcified plaque (43), revealing that AI-assisted CTA has a higher diagnostic performance in evaluating vessel stenosis.

Significant heterogeneity was found in the results among the included studies. Thus, our results should be interpreted cautiously. The use of a subgroup analysis may explain some of the sources of heterogeneity. After grouping according to whether carotid or coronary plaque was detected, a slight drop in *I*^2^ was observed in the SEN (from 95.61% to 76.57%) and SPE (from 98.02% to 94.80%) in the carotid plaque group. We suspected that this may be because the carotid arteries were thicker in diameter and more superficial relative to the coronary arteries, making plaque lesions and stenosis easier to be detected. A drop in *I*^2^ was observed in SEN (from 95.61% to 84.61%) in the prospective study group, suggesting the study design may also be a source of the heterogeneity. Moreover, multicenter studies had higher SPE and DOR, which indicated larger sample sizes group may have better diagnostic performance; thus, multicenter collaboration needs to be encouraged.

However, there are some limitations in our study. First, the number of studies that met the selection criteria is relatively small and the quality of the eligible studies varied. Second, most of the included studies were from China and the United States, which may lead to heterogeneity. Third, only one article focused on plaque vulnerability assessment, which may be discussed in further reviews. Hence, it is still too early to conclude that the AI-based systems could be applied in diagnosing plaques with high accuracy. More studies will be included in the future to provide more credible conclusion.

## 5 Conclusion

AI-assisted CTA was valuable in the diagnosis and classification of plaque, with the best diagnostic efficacy in evaluating plaque with 70% stenosis.

## Data Availability

The original contributions presented in the study are included in the article/Supplementary Material, further inquiries can be directed to the corresponding authors.
