# Supplementary material for: Diagnostic value of artificial intelligence-assisted CTA for the assessment of atherosclerosis plaque: a systematic review and meta-analysis
Source: Front Cardiovasc Med. 2024 Sep 3;11:1398963. doi: 10.3389/fcvm.2024.1398963 (PMC11405224; doi:10.3389/fcvm.2024.1398963)

Supplementary Materials

Diagnostic value of artificial intelligence-assisted CTA for the assessment of atherosclerosis plaques: a systematic review and meta-a nalysis

Contents

Table S1: Literature searching strategies in PubMed, Embase and Cochrane Library database.

Table S2: Detail characteristics of the included studies.

Table S3: The average score for each criterion of the RQS of all eligible studies.

Table S4. The pooled sensitivity, specificity and AUC of AI-assist CTA in identifying plaques, 50% stenosis, 70% stenosis and calcified plaques.

Figure S1. RQS score of eligible study.

Figure S2. Forest plots and Hierarchical summary receiver operating characteristic curve (SROC) plot for the diagnostic performance of AI-assist CTA in identifying 50% stenosis, 70%.stenosis and calcified plaque.

Table S1: Literature searching strategies in PubMed, Embase and Cochrane Library database.

Databases		Retrieval strategy	Items found
**PubMed**	#1	"Artificial Intelligence"[MeSH Terms] OR "artificial intelligence"[Title/Abstract] OR "computational intelligence"[Title/Abstract] OR "machine intelligence"[Title/Abstract] OR "computer reasoning"[Title/Abstract] OR "AI"[Title/Abstract] OR "computer vision systems"[Title/Abstract] OR "computer vision system"[Title/Abstract] OR "knowledge acquisition"[Title/Abstract] OR "knowledge representation"[Title/Abstract] OR "knowledge representations"[Title/Abstract]	232218
	#2	"Machine Learning"[MeSH Terms] OR "machine learning"[Title/Abstract] OR "transfer learning"[Title/Abstract]	118341
	#3	"Deep Learning"[MeSH Terms] OR "deep learning"[Title/Abstract] OR "hierarchical learning"[Title/Abstract]	50,554
	#4	"Supervised Machine Learning"[MeSH Terms] OR "supervised machine learning"[Title/Abstract] OR "semi supervised learning"[Title/Abstract] OR "semi supervised learning"[Title/Abstract] OR "inductive machine learning"[Title/Abstract] OR "active machine learning"[Title/Abstract] OR "learning from labeled data"[Title/Abstract]	14,460
	#5	"supervised Machine Learning"[MeSH Terms]	797
	#6	#1 OR #2 OR #3 OR #4 OR #5	295823
	#7	"Computer Tomography"[MeSH Terms] OR "computer tomography"[Title/Abstract] OR "CT"[Title/Abstract] OR "CTA"[Title/Abstract] OR "Computed tomography angiography "[Title/Abstract] OR "CTCA"[Title/Abstract] OR "computed tomography coronary angiography"[Title/Abstract]	455281
	#8	"High-risk plaque"[MeSH Terms] OR "high-risk plaque"[Title/Abstract] OR "atherosclerosis"[Title/Abstract] OR "atherosclerosis plaque"[Title/Abstract] OR "plaque"[Title/Abstract] OR "vulnerable plaque "[Title/Abstract] OR "calcified plaque"[Title/Abstract]	224349
	#9	("1965/7/10"[Date - Publication] : "2023/7/10"[Date - Publication])	35754676
	#10	#6 AND #7 AND #8 AND #9	247
**Embase**	#1	supervised machine learning'/exp OR 'artificial intelligence':ab,ti OR 'machine intelligence':ab,ti	41,165
	#2	supervised machine learning'/exp OR 'supervised machine learning':ab,ti OR 'semi supervised learning':ab,ti OR 'semi supervised learning':ab,ti OR 'inductive machine learning':ab,ti OR 'learning from labeled data':ab,ti OR 'active machine learning':ab,ti	6297
	#3	deep learning'/exp OR 'deep learning':ab,ti OR 'hierarchical learning':ab,ti	58232
	#4	artificial intelligence'/exp OR 'artificial intelligence':ab,ti OR 'computational intelligence':ab,ti OR 'machine intelligence':ab,ti OR 'computer reasoning':ab,ti OR 'computer vision systems':ab,ti OR 'computer vision system':ab,ti OR 'knowledge acquisition':ab,ti OR 'knowledge representation':ab,ti OR 'knowledge representations':ab,ti	91362
	#5	machine learning'/exp OR 'machine learning':ab,ti OR 'machine learning':ab,ti	413848
	#6	#1 OR #2 OR #3 OR #4 OR #5	455,708
	#7	computer tomography'/exp	1,401,344
	#8	computer tomography':ab,ti OR 'ct':ab,ti OR 'cta':ab,ti OR 'computed tomography angiography ':ab,ti OR 'ctca':ab,ti OR 'computed tomography coronary angiography':ab,ti	758618
	#9	#7 OR #8	
	#10	high-risk plaque'/exp	847
	#11	high-risk plaque':ab,ti OR 'atherosclerosis':ab,ti OR 'atherosclerosis plaque':ab,ti OR 'plaque ':ab,ti OR 'vulnerable plaque':ab,ti OR 'calcified plaque':ab,ti	299330
	#12	#10 OR #11	299,340
	#13	#6 AND #9 AND #12	2,334
**Cochrane Library**	#1	MeSH descriptor: [Artificial Intelligence] explode all trees	2,883
	#2	(Computational Intelligence):ti,ab,kw OR (Artificial Intelligence):ti,ab,kw OR (Computational Intelligence):ti,ab,kw OR (Machine Intelligence):ti,ab,kw OR (Computer Reasoning):ti,ab,kw	1863
	#3	#1 OR #2	4167
	#4	(MeSH descriptor: [Machine Learning] explode all trees	901
	#5	(Machine Learning):ti,ab,kw OR (Transfer Learning):ti,ab,kw	4,029
	#6	#4 OR #5	4,294
	#7	MeSH descriptor: [Deep Learning] explode all trees	272
	#8	(Deep Learning):ti,ab,kw OR (Hierarchical Learning):ti,ab,kw	1591
	#9	#7 OR #8	1591
	#10	#3 OR #6 OR #9	8136
	#11	MeSH descriptor: [Computer Tomography] explode all trees	0
	#12	(Computer Tomography):ti,ab,kw OR (CT):ti,ab,kw OR (CTA):ti,ab,kw OR (Computed Tomography Angiography):ti,ab,kw OR (CTCA):ti,ab,kw OR (Computed Tomography Coronary Angiography):ti,ab,kw	90,677
	#13	#11 OR #12	90,677
	#14	MeSH descriptor: [High-risk Plaque] explode all trees	0
	#15	(High-risk Plaque):ti,ab,kw OR (Atherosclerosis):ti,ab,kw OR (Atherosclerosis Plaque):ti,ab,kw OR (Plaque):ti,ab,kw OR (Vulnerable Plaque):ti,ab,kw OR (Calcified Plaque):ti,ab,kw	28714
	#16	#14 OR #15	28714
	#17	#10 AND #13 AND #16	30

Table S2: Detail characteristics of the included studies.

Author	Year	Country	Mean or Median age	Patients	sex（F/M）	TP	FP	FN	TN	Imaging modality	No of patient	lesions	AUC	Study Design
Acharya	2019	Singapore	60.7 ± 10.4	73	36/37	1945	176	173	904	CCTA	73	2646	-	retrospectively
Han	2020	China	64.0	50	21/29	61	59	34	639	CCTA	50	793	0.75	retrospectively
Li	2021	China	53.0 ± 9.0	36	4/32	175	29	25	121	CCTA	36	350	0.9	retrospectively
Choi	2021	USA	60.0 ± 12.0	232	86/146	11	0	1	220	CCTA	232	232		prospectively + retrospectively
Choi	2021	USA	60.0 ± 12.0	232	86/146	27	6	7	192	CCTA	232	232		prospectively + retrospectively
Choi	2021	USA	60.0 ± 12.0	232	86/146	12	2	1	909	CCTA	232	924		prospectively + retrospectively
Choi	2021	USA	60.0 ± 12.0	232	86/146	35	15	11	863	CCTA	232	924		prospectively + retrospectively
Xu	2021	China	65.7 ± 10.1	306	140/166	342	93	75	714	CCTA	306	1224		retrospectively
Xu	2021	China	65.7 ± 10.1	306	140/166	143	37	72	972	CCTA	306	1224		retrospectively
Xu	2021	China	65.7 ± 10.1	306	140/166	86	120	6	94	CCTA	306	306		retrospectively
Yi	2021	China	63.3 ± 10.7	71	19/52	26	1	3	34	CCTA	71	107		retrospectively
Lin	2022	USA	-	100	-	20	3	2	125	CCTA	100	150	-	prospectively + retrospectively
Lin	2022	USA	-	100	-	55	8	1	86	CCTA	100	150	-	prospectively + retrospectively
Griffin	2022	USA	64.0 ± 10.0	303	85/218	190	32	12	69	CCTA	303	303	0.88	retrospectively
Griffin	2022	USA	64.0 ± 10.0	303	85/218	112	33	7	151	CCTA	303	303	0.95	retrospectively
Cilla	2022	Italy	73.0	30	11/19	16	3	0	11	CTA	30	30	0.96	retrospectively
Hu	2022	China	-	141	57/84	74	9	0	58	CCTA	141	141	-	retrospectively
Fu	2023	China	61.0 ± 11.0	142	63/79	205	32	42	1568	CTA	142	1847	0.91	prospectively + retrospectively
Fu	2023	China	61.0 ± 11.0	142	63/79	405	70	44	1328	CTA	142	1847	0.93	prospectively + retrospectively
Fu	2023	China	61.0 ± 11.0	142	63/79	85	10	8	39	CTA	142	142	0.86	prospectively + retrospectively
Fu	2023	China	61.0 ± 11.0	142	63/79	114	4	6	20	CTA	142	142	0.88	prospectively + retrospectively

Table S3: The average score for each criterion of the RQS of all eligible studies.

Study ID	Acharya	Han	Li	Choi	Xu	Yi	Lin	William	Cilla
Image protocol	1/1	0/0	1/1	1/1	1/1	1/1	1/1	1/1	1/1
Multiple segmentations	0/0	0/0	0/0	0/0	0/0	0/0	0/0	0/0	0/0
Phantom study on all scanners	0/0	0/0	0/0	0/0	0/0	0/0	0/0	0/0	0/0
Multiple time points	0/0	0/0	0/0	0/0	0/0	0/0	0/0	0/0	0/0
Feature reduction	3/3	-3/-3	-3/-3	-3/-3	-3/-3	-3/-3	-3/-3	-3/-3	3/3
Multivariable analysis	0/0	0/0	0/0	0/0	0/0	0/0	0/0	0/0	0/0
Biological correlates	0/0	0/0	0/0	0/0	0/0	0/0	0/0	0/0	0/0
Cut-off	1/1	0/0	1/1	1/1	1/1	1/1	1/1	1/1	1/1
Discrimination	1/1	2/2	2/2	2/2	2/2	2/2	2/2	2/2	2/2
Calibration	0/0	0/0	0/0	0/0	0/0	0/0	0/0	0/0	0/0
Prospective study	0/0	0/0	0/0	7/7	0/0	0/0	0/0	0/0	7/7
Validation	-5/-5	-5/-5	2/2	-5/-5	-5/-5	-5/-5	3/3	-5/-5	-5/-5
Comparison to ‘gold standard’	2/2	2/2	2/2	2/2	2/2	2/2	2/2	2/2	2/2
Potential clinical utility	2/2	2/2	0/0	0/0	2/2	2/2	2/2	0/0	2/2
Cost-effectiveness analysis	0/0	0/0	0/0	0/0	0/0	0/0	0/0	0/0	0/0
Open science	3/3	3/3	2/2	3/3	3/3	3/3	3/3	3/3	3/3
Total	8/8	1/1	7/7	8/8	3/3	1/1	11/11	1/1	16/16

Table S4: The pooled sensitivity, specificity and AUC of AI-assist CTA in identifying plaques, 50% stenosis, 70% stenosis and calcified plaques.

	**Sensitivity**	**Specificity**	**PPV**	**NPV**	**AUC**	**DOR**	**Pubbias**
All study	0.90 (0.85,0.93)	0.93 (0.87,0.96)	12.9 (7.1,23.5)	0.11 (0.08,0.16)	0.96 (0.94-0.97)	116 (61,222)	0.12
50%	0.90 (0.84,0.94)	0.89 (0.76,0.96)	8.4 (3.7,19.0)	0.11 (0.07,0.17)	0.95 (0.93-0.96)	76 (36, 161)	0.98
70%	0.87 (0.78,0.93)	0.98 (0.91,0.99)	37.8 (9.4,151.2)	0.13 (0.07,0.23)	0.96 (0.94-0.97)	294 (65, 1343)	0.29
Calcium	0.94 (0.73,0.99)	0.87 (0.81,0.92)	7.4 (4.9,11.0)	0.06 (0.01,0.36)	0.92 (0.90-0.94)	116 (20, 681)	0.49

Fig S1: RQS score of eligible study.

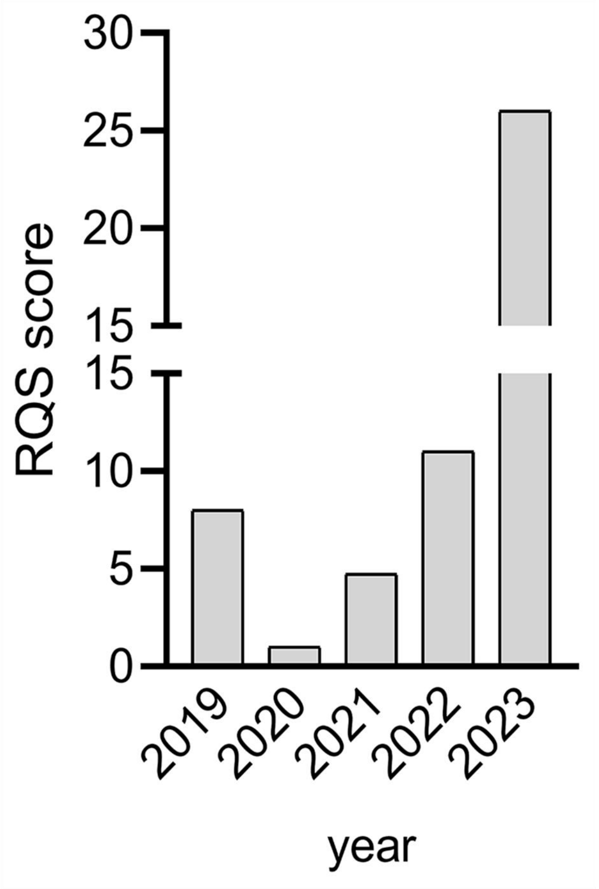

Fig S2: (a, c, e) Forest plots of the pooled sensitivity (left) and specificity (right) for the diagnostic performance of AI-assist CTA in identifying 50% stenosis, 70%.stenosis and calcified plaque. (b, d, f) Hierarchical summary receiver operating characteristic curve (SROC) plot for the diagnostic performance of AI-assist CTA in identifying 50% stenosis, 70%.stenosis and calcified plaque.

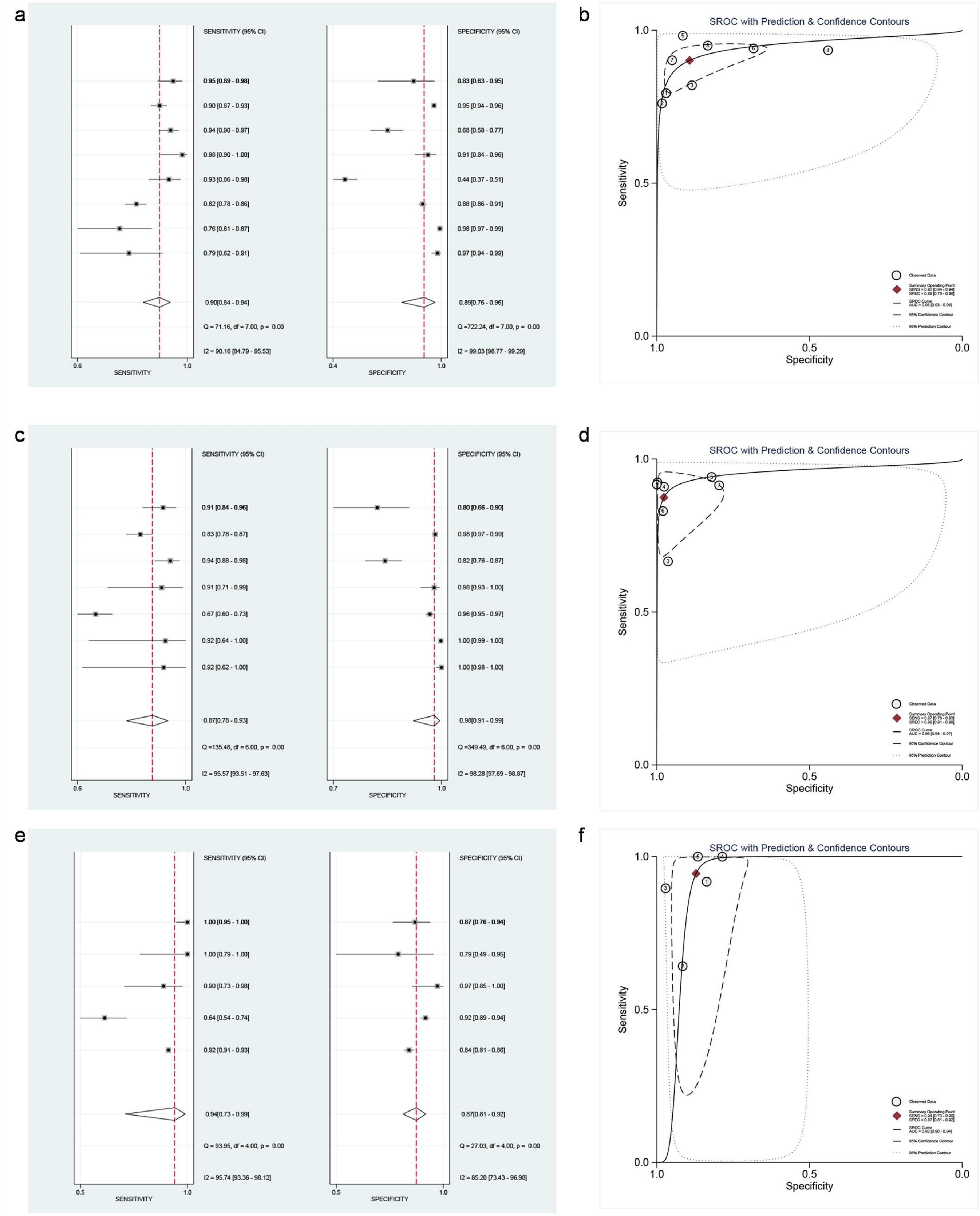

Supplement: Supplementary file 1 [file Datasheet1.pdf]
